# Ferroptosis, Metabolic Rewiring, and Endometrial Cancer

**DOI:** 10.3390/ijms25010075

**Published:** 2023-12-20

**Authors:** Eglė Žalytė

**Affiliations:** Institute of Biosciences, Life Sciences Center, Vilnius University, Saulėtekio av. 7, LT-10257 Vilnius, Lithuania; egle.zalyte@gf.vu.lt; Tel.: +370-5-239-8229

**Keywords:** ferroptosis, metabolism, endometrial cancer, resistance

## Abstract

Ferroptosis is a newly discovered form of regulated cell death. The main feature of ferroptosis is excessive membrane lipid peroxidation caused by iron-mediated chemical and enzymatic reactions. In normal cells, harmful lipid peroxides are neutralized by glutathione peroxidase 4 (GPX4). When GPX4 is inhibited, ferroptosis occurs. In mammalian cells, ferroptosis serves as a tumor suppression mechanism. Not surprisingly, in recent years, ferroptosis induction has gained attention as a potential anticancer strategy, alone or in combination with other conventional therapies. However, sensitivity to ferroptosis inducers depends on the metabolic state of the cell. Endometrial cancer (EC) is the sixth most common cancer in the world, with more than 66,000 new cases diagnosed every year. Out of all gynecological cancers, carcinogenesis of EC is mostly dependent on metabolic abnormalities. Changes in the uptake and catabolism of iron, lipids, glucose, and glutamine affect the redox capacity of EC cells and, consequently, their sensitivity to ferroptosis-inducing agents. In addition to this, in EC cells, ferroptosis-related genes are usually mutated and overexpressed, which makes ferroptosis a promising target for EC prediction, diagnosis, and therapy. However, for a successful application of ferroptosis, the connection between metabolic rewiring and ferroptosis in EC needs to be deciphered, which is the focus of this review.

## 1. Introduction

Ferroptosis is an iron-dependent mode of cell death that is mechanistically different from apoptosis, necroptosis, pyroptosis, and other regulated cell death types. Discovered in 2012 by Dixon et al., ferroptosis quickly gained attention in the context of cardiovascular, neurodegenerative diseases, and cancer [1]. One of the hallmarks of ferroptosis is membrane lipid peroxidation. Lipid peroxides and peroxyl radicals are constantly produced as byproducts of normal cellular homeostasis in Fenton reactions and by enzymes that use iron as a cofactor, such as lipoxygenases. Typically, toxic oxidized lipid species are neutralized by an enzyme glutathione peroxidase 4 (GPX4). When GPX4 is inactivated, ferroptosis occurs [2]. Many of the currently used ferroptosis inducers deplete cells of cysteine, inhibit GPX4 enzymatic activity, or lower GPX4 protein level, whereas ferroptosis is inhibited by lipophilic antioxidants and iron chelators [3]. However, recently, additional proteins (FSP1, DHODH, and GCH1) responsible for the defense against ferroptosis have been identified [4,5,6,7]. Also, although at first plasma membrane was believed to be the main site of lipid peroxidation, recent evidence shows that endoplasmic reticulum (ER) [8], mitochondria [4], lysosomes [9], Golgi complex [10], and peroxisomes [11] are involved as well. It has been shown that certain conditions predispose cells to ferroptosis. For example, mesenchymal cells are more sensitive to ferroptosis induction than epithelial cells due to an increased amount of easily oxidized polyunsaturated fatty acids in cellular membranes [12]. In detached cells, a loss of α6β4 integrin signaling promotes ferroptosis by upregulation of ACSL4 and a decreased expression of GPX4 [13]. Thus, ferroptosis might be a potential strategy to eliminate metastatic cancer cells. In addition, immunotherapy-resistant dedifferentiated melanoma cell subtypes exhibit vulnerability to ferroptosis due to a decreased level of reduced glutathione [14]. A reduced level of glutathione and NADPH was also observed in drug-resistant tumor cell populations, thus making them a target of ferroptosis-inducing agents [15]. Finally, cancer stem cells have an increased labile iron pool in their cytosol and are very susceptible to ferroptosis [16]. Thus, ferroptosis can be used to eliminate tumor cell subpopulations responsible for cancer recurrence. To increase the anticancer effect, ferroptosis can be coupled with chemotherapy [17], radiation therapy [18], photodynamic therapy [19], and, being an immunogenic cell death, with immunotherapy [20].

Endometrial carcinoma (EC) is the sixth most common cancer among women worldwide [21]. Carcinogenesis of EC is highly dependent on metabolic factors. Three metabolic abnormalities, namely, obesity, hyperglycemia, and hormone exposure, increase the risk of EC [22]. Metabolic rewiring is a common trait of cancer cells. Not only does it make them addicted to certain nutrients, such as glucose and glutamine, but it also influences cellular antioxidant defense [23,24,25,26]. Consequently, cell sensitivity to ferroptosis is altered. Thus, the outcome of therapeutic strategies involving ferroptosis inducers critically depends on the metabolic state of the cell. In comparison to normal tissue, EC genes of ferroptosis-related proteins and lncRNAs are mutated, epigenetically modified, and, as a result, differentially expressed. Commonly, a higher expression of ferroptosis genes correlates positively with immune cell infiltration into tumor mass, highlighting the importance of ferroptosis immunogenicity. Not surprisingly, ferroptosis gene signatures are used to create prognostic models for EC patient survival [27].

This review summarizes the current knowledge of ferroptosis, metabolic rewiring in cancer cells, and its relationship to ferroptosis sensitivity, with a particular focus on EC.

## 2. Discovery of Ferroptosis

**Figure 1:**
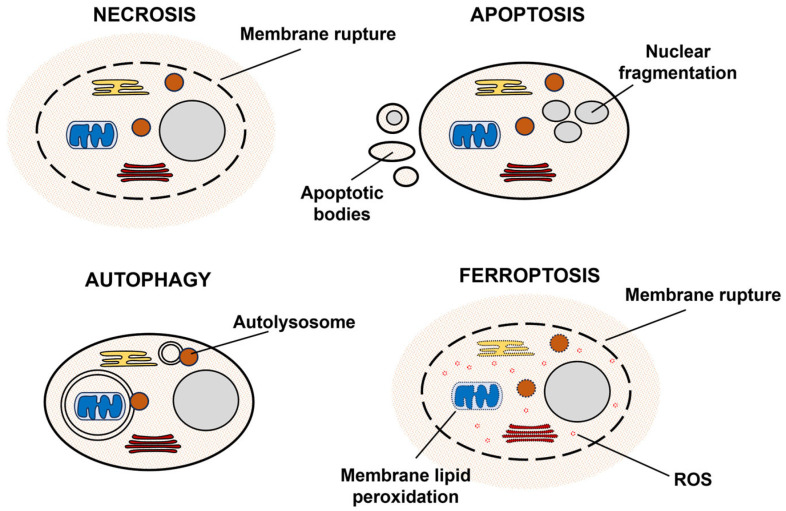
A scheme illustrating different modes of cell death.

Cell death is the inevitable fate of all living organisms. For a long time, the classification of cell death was based solely on morphologic characteristics. Three cell death types were defined: (a) apoptosis, accompanied by rounding up and shrinkage of the cell, retraction of pseudopodia, chromatin condensation, and fragmentation of the nucleus; (b) autophagy, characterized by the formation of autophagosomes; and (c) necrosis, that results in cell swelling and the rupture of the plasma membrane (Figure 1) [28]. Although morphologic classification is still widely used, cell death can also be broadly divided into accidental and regulated. According to the Nomenclature Committee on Cell Death, accidental cell death is induced by various external stresses: extreme temperatures, pH variations, and osmotic and mechanical shock. In contrast, regulated cell death is governed by molecular mechanisms and, therefore, can be intervened by pharmacologic and genetic approaches. When regulated cell death accompanies natural physiological processes (embryogenesis, tissue homeostasis, immune reaction), it is called programmed cell death. However, environmental stress can also induce regulated cell death [29]. According to biochemical processes and morphological features of programmed cell death, it is further divided into twelve types: extrinsic and intrinsic apoptosis, immunogenic cell death, autophagy-dependent cell death, lysosome-dependent cell death, netotic cell death, entotic cell death, parthanatos, necroptosis, mitochondrial permeability transition-driven necrosis, pyroptosis, and ferroptosis [30]. New cell death types, such as methuosis [31], paraptosis [32], autosis [33], alkaliptosis [34], oxeiptosis [35], cuproptosis [36], and erebosis [37], are also emerging.

In 2003, while screening the library of new antitumor agents, Dolma et al. discovered a compound that was selectively lethal to cancer cells expressing oncogenic RAS and Small T oncoprotein. The compound was named erastin (eradicator of RAS and ST) [38]. Interestingly, cell death induced by erastin did not exhibit any biochemical and morphological features of apoptosis and could not be inhibited by apoptosis inhibitors. Eventually, it was shown that this atypical cell death could be prevented by iron chelators. Other compounds, RSL3 (RAS Selective Lethal 3) and RSL5 (RAS Selective Lethal 5), with similar properties to erastin, were discovered in 2008 [39]. RSL3 induced ROS accumulation inside the cell [40]. In the same year, Seiler et al. discovered that inactivation of GPX4 drives lipid peroxidation that can be suppressed by antioxidant α-tocopherol or by inhibition of lipoxygenases [41]. In addition, xCT was identified as a mediator of oxidative stress resistance upon GSH depletion [42]. In 2012, Dixon et al. named the new cell death type “ferroptosis”, based on the Latin ferrum meaning “iron” and Greek ptôsis meaning “fall” [43]. More ferroptosis-mediating compounds, including ferroptosis inducers sulfasalazine, sorafenib, FIN56, FINO2, and inhibitors ferrostatin-1 and liprostatin-1, were discovered in several years [44]. [43]. New antioxidant systems, FSP1, DHODH, and GCH1, which are responsible for ferroptosis resistance, have been actively studied recently [5,6,7]. However, it is worth mentioning that features of ferroptotic cell death had been noticed earlier by other researchers. For example, when neurons are deprived of cysteine, reduced GSH synthesis leads to oxidative stress and cell death, called oxytosis [45]. Oxytosis and ferroptosis are similar from a mechanistic perspective, and sometimes the terms are used interchangeably [46]. However, in the context of cancer, the term ferroptosis is more relevant. Also, there are some differences between oxytosis and ferroptosis; for example, oxytosis is strongly dependent on the uptake of calcium [47], while ferroptosis is caused by cellular iron accumulation [43].

## 3. Inducers and Inhibitors of Ferroptosis

**Figure 2:**
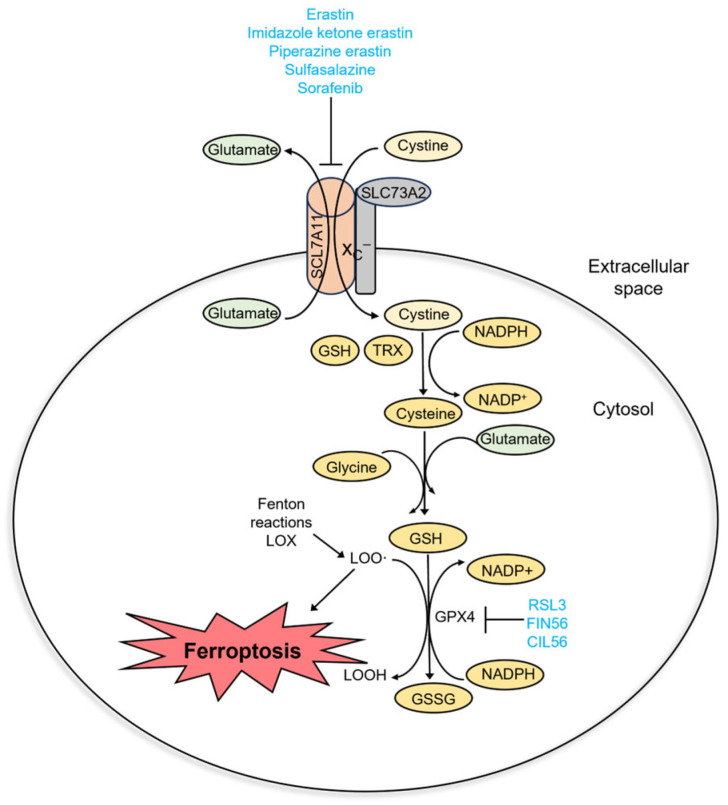
Molecular pathway of ferroptosis.

**Figure 3:**
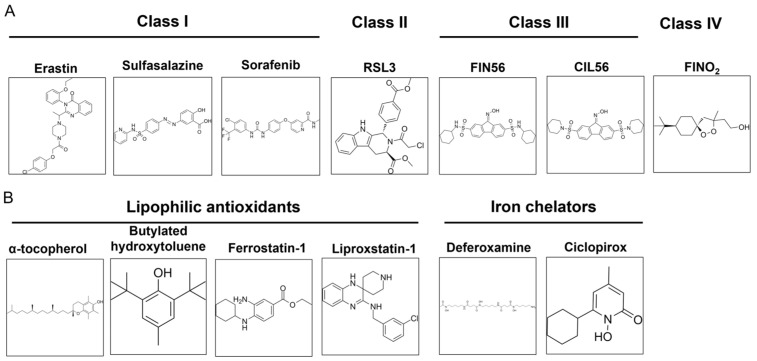
Commonly used ferroptosis inducers (**A**) and inhibitors (**B**).

Ferroptosis is a regulated cell death that is caused by excessive membrane lipid peroxidation [2]. Normally, the oxidative damage of the cell membrane is prevented by glutathione peroxidase 4 (GPX4). Ferroptosis occurs when GPX4 becomes dysfunctional (Figure 2). It can be caused by several factors: inactivation of GPX4 due to GSH depletion, reduced GPX4 activity, and reduced GPX4 protein level. Based on this, four classes of ferroptosis inducers are distinguished (Figure 3A). Class I ferroptosis inducers inhibit cystine/glutamate antiporter x_C_^−^. x_C_^−^ is a transmembrane protein that exchanges one molecule of glutamate to one molecule of cystine, an oxidized form of cysteine. The incorporation of cysteine into GSH is a rate-limiting step of GSH synthesis. When x_C_^−^ is inhibited, the cell is deprived of GSH, and GPX4 activity is reduced. Consequently, oxidative damage to cell membranes is no longer prevented [48]. The main class I ferroptosis inducers are erastin and its analogs imidazole ketone erastin and piperazine erastin. In comparison to erastin, imidazole ketone erastin and piperazine erastin have better water solubility and have more potential to be used in vivo [49]. In addition, class I ferroptosis inducers include repurposed drugs sulfasalazine [50] and sorafenib [51]. Class II ferroptosis inducers inhibit the enzymatic activity of GPX4. One of the most widely used class II ferroptosis inducers is RSL3, which covalently binds to selenocysteine in the active center of GPX4 [52]. Class III ferroptosis inducers, FIN56 (Ferroptosis Inducer 56) and CIN56 (Caspase-Independent Lethal 56), lower the protein level of GPX4 (indirectly activate its degradation) and inhibit the synthesis of endogenic lipophilic antioxidant coenzyme Q [53,54]. Class IV ferroptosis inducers, such as FINO_2_ oxidize iron, indirectly inhibit GPX4 enzymatic activity and induce lipid peroxidation [55]. Out of the mentioned compounds, only imidazole ketone erastin, piperazine erastin, FIN56, sulfasalazine, and sorafenib are suitable for clinical use. However, unlike erastin, its analogs, RSL3 and FIN56, sulfasalazine, and sorafenib, are not specific ferroptosis inducers [56,57].

As ferroptosis is an iron-dependent form of cell death, it can be inhibited by iron chelators (deferoxamine, ciclopirox) and lipophilic antioxidants (α-tocopherol, butylated hydroxytoluene, ferrostatin-1, and liproxstatin-1) (Figure 3B). Iron chelators prevent lipid peroxidation by inhibiting lipoxygenases and Fenton reactions, while lipophilic antioxidants neutralize lipid radicals [48]. The third type of ferroptosis inhibitors are deuterated polyunsaturated fatty acids (D-PUFA, for example, D_4_-arachidonic acid) that stop the initiation and propagation steps of membrane lipid peroxidation. Ferroptosis is also suppressed by inhibitors of lipoxygenases (LOX). Nonspecific ferroptosis inhibitors include cycloheximide (inhibits translation) [40], β-mercaptoethanol (reduces extracellular cystine to cysteine) [43], dopamine (inhibits GPX4 degradation) [58], GPX4 cofactor selenium [59], and vildagliptin (impedes DPP4-dependent lipid peroxidation) [60].

## 4. Molecular Mechanism of Ferroptosis

Mammalian cell membranes are composed of phospholipids, acylated with at least one polyunsaturated fatty acid (PUFA). In contrast to saturated and monounsaturated fatty acids, PUFA bisalylic hydrogen atoms are easily oxidized by free radicals formed in Fenton reactions and by enzymes that use iron as a cofactor: cyclooxygenases, cytochrome P450, and lipoxygenases [61]. Out of the three, lipoxygenases are mostly studied in the context of ferroptosis, and their inhibition suppresses ferroptosis in cancer cells. However, the impact of Fenton and enzymatic reactions is hardly discriminated in ferroptosis, as inhibitors of lipoxygenases also scavenge peroxyl radicals that are formed in Fenton reactions [62].

**Figure 4:**
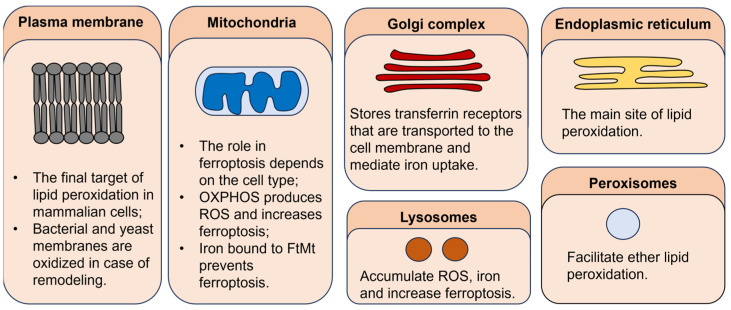
The role of different cellular organelles in ferroptosis.

Although the plasma membrane was believed to be the primary target of lipid peroxidation, recent evidence shows that the rupture of the plasma membrane might be the final step of ferroptosis rather than the first. Oxidation of other organelles, such as endoplasmic reticulum (ER), mitochondria, lysosomes, Golgi complex, and peroxisomes, might be more important in the initiation and propagation of ferroptosis (Figure 4). According to some authors, ER is believed to be the main site of lipid peroxidation. This is supported by the fact that ferroptosis inhibitors primarily accumulate in ER. Changes in ER morphology, such as increased viscosity, are observed during ferroptosis [2]. However, mitochondria are a primary source of ROS in the cell. During ferroptotic cell death, a decrease in mitochondria cristae is present, as well as the rupture of the mitochondrial outer membrane [63]. However, it seems that the role of mitochondria in ferroptosis is cell-type-dependent. For example, cancer cells lacking functional mitochondria are also capable of RSL-3-induced cell death, which indicates that mitochondria are not crucial for ferroptosis [64]. However, in neuronal cells and mouse embryonic fibroblasts, mitochondrial ROS scavenging and preservation of mitochondrial function abolish RSL-3-induced cell death even when plasma membrane lipid peroxidation is evident [65]. Mitochondria also accumulate iron in the form of mitochondrial ferritin (FtMt). Overexpression of FtMt reduces the labile iron pool and prevents ferroptosis in neuroblastoma cells [66]. Lysosomes accumulate ROS and iron as well as mitochondria. Iron overload leads to lysosome membrane oxidation and induces ferroptosis and lysosome-dependent cell death [9]. Also, ferritin and GPX4 degradation in the lysosomes increases the level of ROS in the cell [67]. [64]. Golgi complex and peroxisomes might stimulate iron uptake and ferroptosis by accumulating the transferrin receptor that is transported to the cell membrane and peroxidation of ether lipids, respectively [10,11].

In addition, cell death can be triggered not only by the membrane oxidation itself but also by the toxic side products of lipid peroxidation. Malondialdehyde and 4-hydroxinonenal are toxic byproducts of lipid peroxidation that damage cell proteins and DNA [68,69].

Four cellular antioxidant systems take part in the defense against ferroptosis: GPX4, FSP1, DHODH, and GCH1. The main enzyme responsible for the neutralization of harmful lipid peroxides is GPX4 [70]. GPX4 uses two cofactors: selenocysteine and GSH. In the first step of the GPX4 reaction, selenocysteine attacks lipid peroxide; selenic acid and a neutral lipid hydroxide are formed. The active site of GPX4 is regenerated by two molecules of GSH that reduce selenic acid to selenocysteine [61]. Some cancers, like diffuse large B cell lymphomas and renal cell carcinomas, are very sensitive to GPX4-mediated ferroptosis, whereas others do not require functional GPX4 to survive, mostly due to upregulated antioxidant defense and decreased amount of fatty acids in cellular membranes [71]. Two other proteins, FSP1 (Ferroptosis Suppressor Protein 1), previously known as AIFM2 (Apoptosis-Inducing Factor Mitochondrial 2), and DHODH, prompt coenzyme Q regeneration and inhibit ferroptosis as well [4,5,6]. The third ferroptosis suppressor is GTP cyclohydrolase 1 (GCH1). GCH1 synthesizes tetrahydrobiopterin (BH_4_), a cofactor used by NO synthases (NOS) [72]. An increased level of BH_4_ leads to NOS activation and attenuation of ROS. Not surprisingly, GCH1 expression negatively correlates with cancer cell sensitivity to ferroptosis [7].

## 5. Ferroptosis and Immunity

It has been shown that the cells undergoing ferroptosis secrete DAMPs (damage-associated molecular patterns) and other molecules that attract immune cells and elicit immune responses. Thus, ferroptosis is an immunogenic cell death. This additional tumor suppression mechanism could be successfully exploited in immunotherapy [20]. For example, nanoparticles charged with RSL-3 not only initiated T lymphocyte activation and antitumoral response but also increased resistance to lipid damage repair in tumor cells, leading to accumulation of ROS [73] and sensitivity to radiotherapy [74]. Upon ferroptosis induction, murine fibrosarcoma and glioma cells secrete DAMPs ATP and HMGB1 in vitro and in vivo and promote the maturation of dendritic cells. Some of these DAMPs, such as HMGB1, can further promote ferroptosis. However, studies showed that only early but not late ferroptotic cells display immunogenic properties [75]. On the contrary, another research indicated that early ferroptotic cells decrease antigen cross-presentation in dendritic cells and suppress the proliferation of cytotoxic T lymphocytes [76]. This means that the immunogenicity of ferroptotic cells is time and condition-dependent. In addition, the cells undergoing ferroptosis accumulate oxidized phosphatidylethanolamine and its derivatives, which are the main “eat me” signals for macrophages [77,78]. Dying cells also release prostaglandins, eicosanoids (5-hydroxyeicosatetraenoic acid (5-HETE), 12-HETE, and 15-HETE), and oxidized amino acids that attract immune cells [79]. Lipid oxidation itself can also negatively impact the maturation of dendritic cells [80] and the antitumor properties of CD8^+^ T cells [81]. For example, in pancreatic cancer, ferroptotic cells release 8-hydroxy-2′-deoxyguanosine and activate interferon signaling in tumor-associated macrophages to promote their activation and cancer initiation [82]. An interesting mechanism was proposed by Dai et al., in which pancreatic cancer cells undergoing autophagy-dependent ferroptosis secreted exosomes containing oncogenic KRAS protein that induced macrophage polarization and elicited tumor-promoting effect [83]. Overall, these results indicate that ferroptosis can either induce or suppress immune response. This must be considered when ferroptosis induction is combined with immunotherapeutic agents as antitumor therapy [84].

## 6. The Nature of Ferroptosis

To understand the nature of ferroptosis, we must investigate similar cell death types in evolutionarily distant species. Although all cell membranes are composed of phospholipids (PL), not all PLs contain polyunsaturated fatty acids, which are the main target of ROS. For example, in the membranes of archaea and bacteria, saturated and monounsaturated fatty acids are abundant. The unique composition of cellular membranes allows microorganisms to survive harsh environmental conditions—extreme pH, temperature, and pressure [85]. Thus, bacteria and archaea are usually resistant to ferroptosis. However, some bacteria like *Shewanella* and *Vibrio* utilize polyunsaturated fatty acids to increase membrane fluidity, biofilm formation, and resistance to antibiotics [86,87]. Yeast, as well as bacteria, typically do not synthesize polyunsaturated fatty acids but can accumulate them from the environment and integrate them into their membranes. It was shown that *Saccharomyces cerevisiae* growing in a medium containing polyunsaturated fatty acids are susceptible to oxidative stress [88]. Thus, most likely, ferroptosis is not innate to microorganisms. However, susceptibility to ferroptosis can be gained in certain stressful conditions that require membrane remodeling.

ROS signaling is highly expressed in plants. When *Arabidopsis thaliana* roots are exposed to 55 °C temperature, cell death occurs. It can be prevented by cyclopyrox, ferrostatin-1, or by immersing roots into the solution supplemented with deuterated fatty acids, whose bisalylic carbon cannot be readily oxidized [89]. Although ROS mediate signaling in plants, oxidative stress can be detrimental to plant cells. On the other hand, ferroptosis can be utilized to eliminate the cells that have ROS overload.

In mammalian cells, ferroptosis functions as a mechanism of tumor suppression. It was demonstrated that cancer cells possessing a defective p53 (p533KR) are susceptible to ferroptosis, although cell cycle progression, senescence, and apoptosis are defective. In these cells, the level of SLC7A11, a component of cystine/glutamate antiporter x_C_^−^, is decreased [90]. Additional p53 point mutation K98 eliminates the suppressor function [91]. Tumor suppressor BAP1 (BRCA1-associated protein 1) promotes ferroptosis and inhibits tumor development via a similar mechanism [92]. The third tumor suppressor linked to ferroptosis is fumarase. Fumarase converts fumarate to malate. Inhibition of fumarase leads to fumarate build-up, slowing down the tricarboxylic acid cycle and oxidative phosphorylation. Inactive mitochondria produce less ROS, and ferroptosis is inhibited. When fumarase is active, oxidative phosphorylation progresses and ferroptosis is initiated [93]. Overall, ferroptosis is a rather simple process that does not require sophisticated signaling, transcription, or translation steps. Thus, ferroptosis could be a form of natural selection regulated by the level of only one amino acid, cysteine, in the cellular microenvironment [94]. Furthermore, unlike apoptosis, ferroptosis is an immunogenic cell death and could be used to induce an immune response to eliminate tumors or pathogen infections [95].

## 7. Detection of Ferroptosis: Markers and Methods

To date, the best-studied genetic markers of ferroptosis are *PTGS2* (prostaglandin-endoperoxide synthase 2), *CHAC1* (glutathione specific gamma-glutamylcyclotransferase 1), *SCL7A11* (a component of system x_C_^−^), *ACSL4* (fatty acid-CoA ligase 4), and *RGS4* (regulator of G protein signaling 4). Except for *RGS4*, the expression of all these genes is upregulated in cells undergoing ferroptosis [2]. However, the database of ferroptosis regulators and markers FerrDb V2 (http://www.zhounan.org/ferrdb/, accessed on 6 November 2023) currently annotates nearly fifty positive and negative markers of ferroptosis, i.e., proteins and non-coding RNAs, whose expression changes after ferroptosis induction (Table 1). Most of these proteins are involved in lipid and iron homeostasis, signal transduction, and the regulation of transcription.

In addition to the detection of gene expression changes, ferroptosis can be monitored by other methods. Cell death after the treatment with ferroptosis inducers can be determined by the loss of integrity of the plasma membrane. Propidium iodide is usually used to determine the proportion of dead cells in the population, as it only stains the cells with ruptured membranes. For the quantification, propidium iodide staining can be coupled with flow cytometry. Also, in parallel, cells can be treated with specific ferroptosis inhibitors to prove that the loss of membrane integrity was caused by ferroptosis and no other types of cell death. Another hallmark of ferroptosis is membrane lipid peroxidation, which can be detected with fluorescent dies that react with lipid peroxides or peroxyl radicals, such as C11-BODIPY, Click-It LAA, and LiperFluo [96]. For example, when fluorescent lipid peroxidation reporter C11-BODIPY 581/591 is oxidized, its fluorescence changes from red to green. A fluorescent microscope or flow cytometer then detects a decrease in red fluorescence intensity (the amount of reduced membrane lipids is decreased) and an increase in green fluorescence intensity (the amount of oxidized membrane lipids is increased) [97]. Also, the oxidation of lipid membranes can be detected indirectly by measuring the formation of the reaction side products, such as malondialdehyde and 4-hydroxynonenal, by LC-MS/MS and with antibodies, for example, anti-HNE FerAb antibody, the HNEJ-1 antibody or the anti-malondialdehyde (MDA) adduct 1F83 antibody [2]. Ferroptosis can be monitored by spectrophotometrically assessing the levels of GSH/GSSG and Fe^2+^/Fe^3+^. New fluorescent probes are being developed for the purpose of imaging ferroptosis in live cells [98,99].

## 8. Starvation-Induced Metabolic Rewiring in Cancer Cells

During carcinogenesis, tumor cells are constantly exposed to starvation. At the beginning of carcinogenesis, the need for nutrients and energy is increased due to rapid cell proliferation. Only the cells that adapt to nutrient deprivation survive. In a growing tumor, nutrient gradients are formed that limit cell capacity to utilize them. During metastasis cancer cells are constantly facing nutrient deprivation in different microenvironment niches. In all these cases, the limitation of nutrients rewires cell metabolism and induces cancer cell addiction to certain types of nutrients [100]. For example, tumor cells become addicted to glutamine, arginine, or serine [101,102,103]. A gain of certain addiction depends on the nutrients in the bloodstream, tissue type, tumor heterogeneity, interactions among cancer and stromal cells, and functional needs of certain types of cancer cells [104]. It is not clear whether metabolic changes required for carcinogenesis are innate or adaptive. On the one hand, primary tumors can be composed of phenotypically identical clones that change with regard to nutrient and oxygen availability. Clones that adapt more rapidly proliferate and metastasize. In other models, all tumor cells exhibit different metabolic phenotypes. Although this metabolic heterogeneity can be useless at the beginning of carcinogenesis, it can be utilized by cancer cells during metastasis formation [105]. Overall, there are two metabolic rewiring types in cancer cells: flexibility, or the ability to use different nutrients for energy production, and plasticity, or the ability to use the same nutrients in different metabolic pathways. Metabolic flexibility is important in the early stages of tumor development, whereas plasticity dominates in metastases [106,107].

## 9. Metabolic Rewiring and Ferroptosis

Ferroptosis-related metabolic rewiring in cancer cells stems from an elevated cystine and NADPH metabolism. Cystine is imported into the cell via cystine/glutamate antiporter x_C_^−^. A transport protein of x_C_^−^, SLC7A11, is overexpressed in many cancers [108]. SLC7A11 exchanges one molecule of glutamate to one molecule of cystine. It is estimated that cells lose thirty to fifty percent of cellular glutamate in exchange for cystine [109]. Inside the cell, cystine is reduced to cysteine by thioredoxin or GSH and incorporated into proteins, new glutathione molecules, or used for the synthesis of taurine and H_2_S. Both thioredoxin and GSH utilize NADPH for cystine reduction. An increased glutamate uptake and NADPH catabolism make cancer cells addicted to other nutrients—glutamine and glucose. Glutamine is mainly transported to the cell via ASCT2. Glutaminase catalyzes the conversion of glutamine to glutamate, which is used for the synthesis of GSH or metabolized to α-ketoglutarate that enters the tricarboxylic acid cycle. In cancer cells overexpressing SLC7A11, glutamine import and the activity of glutaminase must be elevated to compensate for glutamate export and glutamine addiction, which is acquired [23,24]. NADPH is synthesized in a pentose phosphate pathway that utilizes glucose. When cells experience glucose deprivation, NADPH synthesis is halted, and toxic cystine and ROS accumulate inside the cell. This way, SLC7A11 overexpression makes cancer cells addicted to glutamine and glucose [25]. Not surprisingly, cancers with glutamine and glucose addiction are sensitive to class I ferroptosis inducers that inhibit SLC7A11 [110,111]. Vice versa, cells overexpressing SLC7A11 are vulnerable to agents that target glutamine and glucose metabolism, such as inhibitors of glutaminase and glucose transporters [112].

## 10. Metabolic Rewiring in Endometrial Cancer

Endometrial carcinoma (EC) is a cancer type that is caused by a malignant transformation of the epithelial cells in the lining of the uterus. It is the sixth most common cancer among women worldwide [21]. Common risk factors of EC are aging, obesity, prolonged estrogen exposure, family history, and some inheritable diseases, such as Lynch syndrome [113]. In addition, out of all gynecological cancers, EC is mostly related to metabolic abnormalities. The term “triple syndrome of endometrial cancer” refers to three metabolic disorders that increase the risk of EC: obesity, hyperglycemia, and hypertension [22]. Metabolic changes related to obesity and hyperglycemia connect EC to ferroptosis sensitivity.

**Figure 5:**
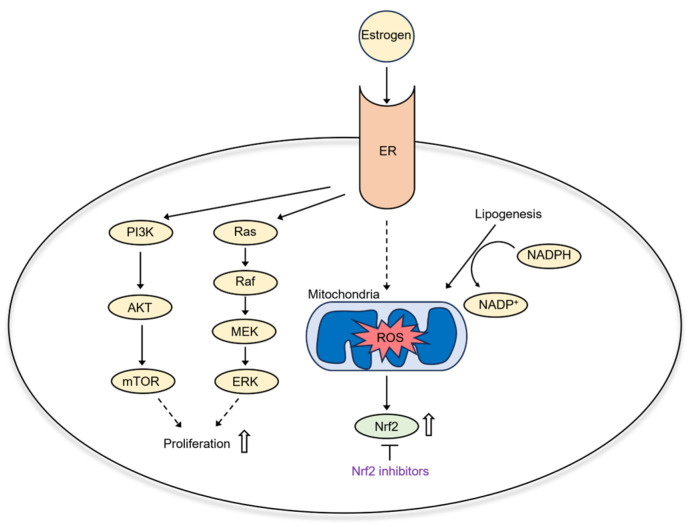
Estrogen signaling in EC.

In post-menopausal women, adipose tissue mediates the conversion of androgens to estrogens [114]. Estrogen binding to its cytoplasmic receptors initiates signaling cascades that stimulate endometrial cancer cell proliferation, such as ETV4 [115], PI3K/Akt, and Ras–Raf–MEK–ERK (Figure 5) [116]. Estrogen is a double-edged sword in oxidative stress: On one hand, it enhances mitochondrial ROS production and activates redox-active transcription factors. On the other hand, estrogen activates antioxidant Nrf2-Keap1 signaling [117]. Thus, estrogen-sensitive cancers like EC have an upregulated antioxidant defense response and can be targeted by antioxidant inhibitors in combination with ROS inducers. A high-fat diet also causes dyslipidemia—changes in lipid metabolism [118]. As lipogenesis utilizes NADPH, a decreased cellular NADPH/NADP+ ratio causes mitochondrial ROS accumulation and oxidative stress [119]. In addition, it was shown that adipose tissue in obese mice accumulates iron [120]. Thus, metabolic changes during obesity may sensitize EC to ferroptosis inducers.

**Figure 6:**
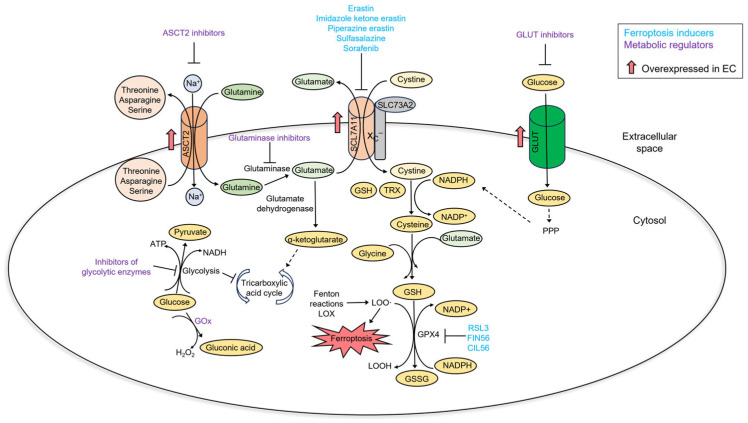
A summary of metabolic changes in EC and their relationship with ferroptosis.

EC exhibits glucose and glutamine dependency, as well as SLC7A11 overexpression [121,122,123]. This relates to EC cell metabolic rewiring and sensitivity to ferroptosis (Figure 6). First of all, EC cells rely on a glycolytic–lipogenic metabolism rather than oxidative phosphorylation for energy production [124]. A high rate of glycolysis inhibits the tricarboxylic acid cycle and decreases the levels of NADH and, consequently, ROS. Inhibiting glycolysis promotes the tricarboxylic acid cycle and oxidative phosphorylation and could sensitize cancer cells to ferroptosis induction [125]. Not surprisingly, therapies targeting glycolysis in EC cells have been investigated. Most of these approaches inhibit glycolytic enzymes [126,127,128]. Glucose concentration within the tumor can be lowered by glucose oxidase-based therapies. Glucose oxidase catalyzes the conversion of glucose to gluconic acid and hydrogen peroxide. Thus, this reaction not only utilizes glucose but also increases ROS [129]. Glucose oxidase can be immobilized on nanoparticles and then specifically targeted to tumor cells, alone or in combination with ferroptosis-inducing compounds [130,131,132]. As glutamine addiction is coupled with sensitivity to ferroptosis, therapies targeted to glutamine metabolism could also be exploited in EC. Indeed, glutamine transporter ASCT2 is upregulated in EC. Inhibition of glutamine uptake resulted in a decreased EC cell proliferation [122]. Also, a recent study showed that inhibition of glutaminase, an enzyme that converts glutamate to glutamine, suppresses EC cell growth in vitro and in vivo [133].

Not surprisingly, other gynecological tumors share similarities with EC. Induction of ferroptosis has been proposed as a possible treatment for ovarian and cervical cancer. For example, ovarian cancer cells tend to accumulate iron due to an increased expression of transferrin receptor I and a decreased expression of ferroportin. Lowering the intracellular iron pool inhibits ovarian cancer cell proliferation in vitro and in vivo and also reduces ovarian cell dissemination [134,135]. Mutations of *TP53* are predominant in ovarian carcinoma [136], and compounds that upregulate p53 increase ovarian cell sensitivity to ferroptosis [137,138]. In addition, erastin was shown to reverse docetaxel resistance in ovarian cancer by reducing the activity of multidrug transporter ABCB1. However, ovarian cancer cells continuously exposed to erastin increase the expression of cysteine biosynthesis and develop resistance [139]. In cervical cancer, ferroptosis can be induced with oleanolic acid, which increases the expression of ACSL4, an enzyme that mediates the synthesis of PUFA-PL [140]. As well as in ovarian cancer, ferroptosis inducers can be exploited to inhibit cervical cancer cell proliferation [141,142]. From a metabolic perspective, as well as EC, ovarian cancer exhibits an increased glucose uptake and a high rate of glycolysis [143,144]. However, not all ovarian cancers are glucose addicted, and the ones dependent on glucose are associated with a better prognosis [145]. Glycolytic enzymes and regulators HK2, PFKFB3, PKM2, and LDH are overexpressed in ovarian cancer and contribute to ovarian carcinogenesis and resistance to chemotherapeutics [146,147,148,149]. On the other hand, glucose-independent ovarian tumors rely on OXPHOS [150]. Glutamine addiction mediated by an increased expression of glutamine transporter ASCT2 and glutaminase correlates with poor patient survival and resistance to platinum drugs [151]. In cervical cancer, expression of glucose transporter GLUT1 is upregulated in late stages and positively correlates with HPV infection, lower immune cell scores, and metastasis [152]. The main regulators of glucose addiction in cervical cancer are glycolytic enzymes PFKFB3 and FBP [153]. What is more, HPV remodels the metabolism of cervical cancer cells via different mechanisms involving glucose uptake, catabolism, glutaminolysis, and the Warburg effect [154].

## 11. Ferroptosis-Based Prognostic Models of Endometrial Cancer

Wang et al. identified sixty ferroptosis-related genes whose expression was altered in EC versus normal tissues. The authors found out that the upregulated expression of *GPX4*, *SAT1*, and *TP53* correlates with a better prognosis, whereas the upregulated expression of *CBS*, *CHAC1*, and *CISD1* was a prognostic risk factor. Missense mutations were present in the *TP53* gene; *GPX4*, *PGD*, and *CHAC1* bared deletions; in *TFRC*, *KEAP1*, *PHKG2*, and *SQLE*, amplifications were present. The authors divided EC samples into four clusters based on the expression of ferroptosis genes. The clusters exhibited a different tumor-infiltrating cell pattern. A total of 21 types of cells were identified in EC samples, out of which activated CD8 T cells, eosinophils, CD56dim NK cells, and activated B cells correlated with a better EC prognosis. The analysis of differentially expressed ferroptosis genes in four clusters revealed thirteen of them, namely, *TUBB4A*, *TMPRSS2*, *STX18*, *LINC01224*, *SLC25A35*, *CD7*, *COL23A1*, *ZG16B*, *KCNK6*, *NWD1*, *C11orf63*, *GZMM*, and *NMU*, as ferroptosis gene signatures [27]. Although the direct relation of these genes to EC is unclear, some of them are involved in other oncogenic diseases. For example, TMPRSS2 (transmembrane serine protease) is a prognostic biomarker in breast and lung cancer. In addition, its expression positively correlates with immune cell infiltration [155]. STX18 (syntaxin 18) mediates DNA damage response and promotes EMT in lung cancer [156], and LINC01224 (lncRNA 1224) is involved in colorectal cancer progression by sponging of miR-485-5p [157]. The upregulation of ZG16B (Zymogen granule protein 16B) expression correlates to a better prognosis of breast cancer patients [158]; however, ZG16B promotes colorectal cancer progression through the Wnt-β-catenin pathway [159]. KCNK6 (Potassium channel subfamily K member 6) promotes breast cancer cell proliferation and metastasis [160]. NWD1 (NACHT and WD repeat domain-containing 1) modulates androgen receptor signaling in prostate cancer [161]. GZMM (granzyme M) increases chemoresistance and EMT in vitro and metastasis in vivo [162]. NMU (neuromedin U)-expressing macrophages stimulate CRC metastatic potential [163].

Qin et al. analyzed the expression of ferroptosis-related genes in different stages of EC. The authors found out that the expression of *HSPA5*, *HSPB1*, *CS*, *CARS*, *EMC2*, *TFRC*, *NCOA4*, *ACSL4*, *RPL8*, *GPX4*, *CDKN1A*, *LPCAT3*, *NFE2L2*, *CISD1*, *SLC1A5*, *SAT1*, *FDFT1*, and *MT1G* was upregulated, whereas *FANCD2*, *SLC7A11*, *GLS2*, *DPP4*, and *ALOX15* expression were downregulated in grade 1-3 EC. The overexpression of *CDKN1A*, *SLC7A11*, and *SAT1* was a positive prognostic factor of EC. However, overexpression of *ATP5MC3* correlated with a higher EC grade and poor patient survival. SAT1 (spermidine/spermine N1-acetyltransferase 1) is a known regulator of the oxidative stress response; its activation increases ALOX15 expression, promotes lipid peroxidation, and, consequently, ferroptosis [164]. In addition, SAT1 overexpression in tumor tissues correlates with a higher degree of infiltrating macrophages and CD8+ T cells in tumor mass; however, SAT1 prognostic value depends on a tumor type [165].

Another study created a prognostic model for EC that contained six ferroptosis and lipid metabolism-related genes: *CDKN1A*, *ESR1*, *PGR*, *CDKN2A*, *PSAT1*, and *RSAD2*. PSAT1 was confirmed as a modulator of EC cell proliferation and migration. Genes related to lipid metabolism and ferroptosis correlated with a higher EC risk, and the degree of immune cell (B cells, T cells CD8, monocytes) tumor infiltration was higher in a low-risk group. The authors also showed that immune checkpoint proteins CTLA-4 and PD1 are potential targets for low-risk EC patients, while high-risk patients were more likely to benefit from standard chemotherapeutics, such as cisplatin, paclitaxel, a multikinase inhibitor AMG.706 and PARP inhibitor ABT.888. These results highlight the importance of personalized therapy for patients with different EC risk statuses [166].

Recently, several lncRNAs were identified as ferroptosis inhibitors in prostate, lung, and bladder cancer [167,168,169]. Also, it was shown that ferroptosis-related lncRNAs have a prognostic value in ovarian cancer [170]. Authors identified nine lncRNA that modulate ferroptosis resistance and proposed that it could be an independent prognostic index of EC. The knockdown of CFAP58-DT lncRNA decreased the viability and migratory properties of EC cells Ishikawa and HEC-1A. In accordance with the previous studies, low and high-risk EC groups differed in the degree of immune cell infiltration; for example, the number of dendritic cells and T cells was lower in the tissues of high-risk patient tumors [171].

Liu et al. investigated the mutational status and epigenetic modifications of twenty-four ferroptosis-related genes among different cancer types. Interestingly, the mutation rate of thirteen ferroptosis genes in EC was higher than five percent, although in other cancers, except for *NFE2L2*, it was low. The upregulated ferroptosis genes among all cancers were *SLC7A11*, *FANCD2*, *CARS*, *SLC1A5*, and *RPL8*, while the expression of *NCOA4* was downregulated. In most cases, the expression of ferroptosis genes correlated with copy number variations. In addition, most ferroptosis genes had a distinctive promoter methylation pattern among different cancers, and only *CDKN1A* exhibited consistent hypomethylation. However, as expected, in all cases, DNA methylation correlated with a decreased gene expression. Also, the study showed that the expression of ferroptosis genes *ACSL4*, *NCOA4*, and *CDKN1A* can be altered by miRNAs and is tumor-specific [172].

A study by Zhang et al. identified thirteen genes whose expression differed in type I and type II EC: *MAPK1*, *PHLPP1*, *ESR1*, *MDM2*, *CDKN2A*, *CDKN1A*, *AURKA*, *BCL2L1*, *POLQ*, *PIK3R3*, *RHOQ*, *EIF4E*, and *LATS2*. Most of these genes possessed amplifications and were involved in the regulation of cell death and tumor differentiation [173].

## 12. Conclusions

Ferroptosis is a newly discovered cell death type that requires iron overload, oxidation-prone polyunsaturated fatty acids in cellular membranes, and inactive GPX4. However, it is still unknown where exactly in the cell ferroptosis is initiated and executed. Possible targets include endoplasmic reticulum, Golgi complex, mitochondria, lysosomes, and peroxisomes. Also, it is still debated what a critical factor causing cell death is—membrane protrusions caused by lipid peroxidation or toxic side products of the oxidation reaction. Although at first glance, ferroptosis seems to be a rather primitive cell death type, its regulation is complex and depends on many factors, including cell metabolism. Metabolic rewiring is an intrinsic trait of cancer cells; however, not all cancers depend equally on metabolic abnormalities. In EC, many ferroptosis-related genes coding proteins and lncRNAs are mutated and differentially expressed in comparison to normal tissue. Overexpression of some, like SLC7A11, makes EC cells addicted to glucose and glutamine. This way, EC cells become vulnerable to agents that target glutamine and glucose metabolism. However, an elevated glucose and glutamine uptake increase EC cell resistance to oxidative stress and, consequently, ferroptosis-inducing agents. Thus, cell sensitivity to ferroptosis and metabolism are interconnected. This can be exploited by therapies that combine ferroptotic stimuli with metabolism-targeting agents. For example, inhibition of glycolysis or glutamine uptake combined with ferroptosis inducers kills two birds with the same stone and significantly improves the therapeutic effect. Thus, deciphering the connection between ferroptosis and cellular metabolism is important for both scientific research and clinical applications.

## Figures and Tables

**Table 1:** Biomarkers of ferroptosis.

Gene	Gene Product	Expression Change	PMID
*ARRDC3*	Arrestin domain-containing protein 3	↑	24844246
*ASNS*	Asparagine synthetase	↑	24844246
*ATF3*	Cyclic AMP-dependent transcription factor ATF-3	↑	24844246
*ATF4*	Cyclic AMP-dependent transcription factor ATF-4	↑	24844246
*ATP6V1G2*	V-type proton ATPase subunit E	↑	24844246
*BLOC1S5-TXNDC5*	Biogenesis of lysosome-related organelles complex 1 subunit 5	↓	24844246
*CAPG*	Macrophage-capping protein	↓	31108460
*CBS*	Cystathionine beta-synthase	↑	24844246
*CEBPG*	CCAAT/enhancer-binding protein gamma	↑	24844246
*DDIT3*	DNA damage-inducible transcript 3 protein	↑	24844246
*DDIT4*	DNA damage-inducible transcript 4 protein	↑	24844246
*DRD5*	D(1B) dopamine receptor	↑	27793671
*DUSP1*	Dual specificity protein phosphatase 1	↑	24439385
*FTH1*	Ferritin heavy chain	↓	27514700
*GABPB1*	GA-binding protein subunit beta-1	↓	31700067
*GDF15*	Growth/differentiation factor 15	↑	24844246
*GPT2*	G patch domain-containing protein 2	↑	24844246
*GPX4*	Phospholipid hydroperoxide glutathione peroxidase	↓	27773819
*HBA1*	Hemoglobin subunit alpha	↑	31108460
*HERPUD1*	Homocysteine inducible ER protein with ubiquitin-like domain 1	↑	24844246
*HMOX1*	Heme oxygenase 1	↑	26097885
*JDP2*	Jun dimerization protein 2	↑	24844246
*KLHL24*	Kelch-like protein 24	↑	24844246
*LOC284561*	Long non-coding RNA	↑	24844246
*LOC390705*	Long non-coding RNA	↓	24844246
*LURAP1L*	Leucine-rich adaptor protein 1-like	↑	24844246
*MT3*	Metallothionein-3	↑	24439385
*NCF2*	Neutrophil cytosol factor 2	↑	24439385
*NNMT*	Nicotinamide N-methyltransferase	↑	31108460
*NOS2*	Nitric oxide synthase, inducible	↑	24439385
*OXSR1*	Serine/threonine-protein kinase OSR1	↑	24439385
*PCK2*	Phosphoenolpyruvate carboxykinase (GTP)	↑	24844246
*PLIN4*	Perilipin-4	↑	31108460
*PSAT1*	Phosphoserine aminotransferase	↑	24844246
*RGS4*	Regulator of G-protein signaling 4	↓	24844246
*RRM2*	rRNA methyltransferase 2	↓	31108460
*SELENOS*	Selenoprotein S	↑	24439385
*SESN2*	Sestrin-2	↑	24844246
*SLC1A4*	Neutral amino acid transporter A	↑	24844246
*SLC3A2*	Amino acid transporter heavy chain SLC3A2	↑	24844246
*SLC7A5*	Large neutral amino acids transporter small subunit 1	↑	24844246
*SNORA16A*	Small nucleolar RNA	↓	24844246
*SRXN1*	Sulfiredoxin-1	↑	24439385
*STMN1*	Stathmin	↓	31108460
*TRIB3*	Tribbles homolog 3	↑	24844246
*TSC22D3*	TSC22 domain family protein 3	↑	24844246
*TUBE1*	Tubulin epsilon chain	↑	24844246
*TXNIP*	Thioredoxin-interacting protein	↑	24844246
*TXNRD1*	Thioredoxin reductase 1	↑	24439385
*UBC*	Polyubiquitin-C	↑	24439385
*VEGFA*	Vascular endothelial growth factor A	↑	24844246
*VLDLR*	Very low-density lipoprotein receptor	↑	24844246
*XBP1*	X-box-binding protein 1	↑	24844246
*ZFP69B*	Zinc finger protein 69 homolog B	↑	24844246
*ZNF419*	Zinc finger protein 419	↑	24844246

## Data Availability

Not applicable.

## References

[1] Han C., Liu Y., Dai R., Ismail N., Su W., Li B. (2020). Ferroptosis and Its Potential Role in Human Diseases. Front. Pharmacol..

[2] Stockwell B.R. (2022). Ferroptosis turns 10: Emerging mechanisms, physiological functions, and therapeutic applications. Cell.

[3] Du Y., Guo Z. (2022). Recent progress in ferroptosis: Inducers and inhibitors. Cell Death Discov..

[4] Bersuker K., Hendricks J.M., Li Z., Magtanong L., Ford B., Tang P.H., Roberts M.A., Tong B., Maimone T.J., Zoncu R. (2019). The CoQ oxidoreductase FSP1 acts parallel to GPX4 to inhibit ferroptosis. Nature.

[5] Doll S., Freitas F.P., Shah R., Aldrovandi M., da Silva M.C., Ingold I., Goya Grocin A., Xavier da Silva T.N., Panzilius E., Scheel C.H. (2019). FSP1 is a glutathione-independent ferroptosis suppressor. Nature.

[6] Mao C., Liu X., Zhang Y., Lei G., Yan Y., Lee H., Koppula P., Wu S., Zhuang L., Fang B. (2021). Author Correction: DHODH-mediated ferroptosis defense is a targetable vulnerability in cancer. Nature.

[7] Kraft V.A.N., Bezjian C.T., Pfeiffer S., Ringelstetter L., Muller C., Zandkarimi F., Merl-Pham J., Bao X., Anastasov N., Kossl J. (2020). GTP Cyclohydrolase 1/Tetrahydrobiopterin Counteract Ferroptosis through Lipid Remodeling. ACS Cent. Sci..

[8] Yang W.S. (2023). Ferroptosis: whERe is the critical site of lipid peroxidation?. Front. Cell Dev. Biol..

[9] Wang F., Gomez-Sintes R., Boya P. (2018). Lysosomal membrane permeabilization and cell death. Traffic.

[10] Alborzinia H., Ignashkova T.I., Dejure F.R., Gendarme M., Theobald J., Wolfl S., Lindemann R.K., Reiling J.H. (2018). Golgi stress mediates redox imbalance and ferroptosis in human cells. Commun. Biol..

[11] Tang D., Kroemer G. (2020). Peroxisome: The new player in ferroptosis. Signal Transduct. Target. Ther..

[12] Liu H., Schreiber S.L., Stockwell B.R. (2018). Targeting Dependency on the GPX4 Lipid Peroxide Repair Pathway for Cancer Therapy. Biochemistry.

[13] Brown C.W., Amante J.J., Goel H.L., Mercurio A.M. (2017). The alpha6beta4 integrin promotes resistance to ferroptosis. J. Cell Biol..

[14] Tsoi J., Robert L., Paraiso K., Galvan C., Sheu K.M., Lay J., Wong D.J.L., Atefi M., Shirazi R., Wang X. (2018). Multi-stage Differentiation Defines Melanoma Subtypes with Differential Vulnerability to Drug-Induced Iron-Dependent Oxidative Stress. Cancer Cell.

[15] Hangauer M.J., Viswanathan V.S., Ryan M.J., Bole D., Eaton J.K., Matov A., Galeas J., Dhruv H.D., Berens M.E., Schreiber S.L. (2017). Drug-tolerant persister cancer cells are vulnerable to GPX4 inhibition. Nature.

[16] Mai T.T., Hamai A., Hienzsch A., Caneque T., Muller S., Wicinski J., Cabaud O., Leroy C., David A., Acevedo V. (2017). Salinomycin kills cancer stem cells by sequestering iron in lysosomes. Nat. Chem..

[17] Liu X., Zhang Y., Wu X., Xu F., Ma H., Wu M., Xia Y. (2022). Targeting Ferroptosis Pathway to Combat Therapy Resistance and Metastasis of Cancer. Front. Pharmacol..

[18] Lei G., Mao C., Yan Y., Zhuang L., Gan B. (2021). Ferroptosis, radiotherapy, and combination therapeutic strategies. Protein Cell.

[19] Huang Y., Li X., Zhang Z., Xiong L., Wang Y., Wen Y. (2023). Photodynamic Therapy Combined with Ferroptosis Is a Synergistic Antitumor Therapy Strategy. Cancers.

[20] Yin J., Meng X., Peng L., Xie W., Liu X., He W., Li S. (2023). Ferroptosis and Cancer Immunotherapy. Curr. Mol. Med..

[21] Sung H., Ferlay J., Siegel R.L., Laversanne M., Soerjomataram I., Jemal A., Bray F. (2021). Global Cancer Statistics 2020: GLOBOCAN Estimates of Incidence and Mortality Worldwide for 36 Cancers in 185 Countries. CA Cancer J. Clin..

[22] Yang X., Wang J. (2019). The Role of Metabolic Syndrome in Endometrial Cancer: A Review. Front. Oncol..

[23] Muir A., Danai L.V., Gui D.Y., Waingarten C.Y., Lewis C.A., Vander Heiden M.G. (2017). Environmental cystine drives glutamine anaplerosis and sensitizes cancer cells to glutaminase inhibition. eLife.

[24] Timmerman L.A., Holton T., Yuneva M., Louie R.J., Padro M., Daemen A., Hu M., Chan D.A., Ethier S.P., van‘t Veer L.J. (2013). Glutamine sensitivity analysis identifies the xCT antiporter as a common triple-negative breast tumor therapeutic target. Cancer Cell.

[25] Liu X., Olszewski K., Zhang Y., Lim E.W., Shi J., Zhang X., Zhang J., Lee H., Koppula P., Lei G. (2020). Cystine transporter regulation of pentose phosphate pathway dependency and disulfide stress exposes a targetable metabolic vulnerability in cancer. Nat. Cell Biol..

[26] Zalyte E., Cicenas J. (2022). Starvation mediates pancreatic cancer cell sensitivity to ferroptosis via ERK1/2, JNK and changes in the cell mesenchymal state. Int. J. Mol. Med..

[27] Wang H., Wu Y., Chen S., Hou M., Yang Y., Xie M. (2021). Construction and Validation of a Ferroptosis-Related Prognostic Model for Endometrial Cancer. Front. Genet..

[28] Galluzzi L., Maiuri M.C., Vitale I., Zischka H., Castedo M., Zitvogel L., Kroemer G. (2007). Cell death modalities: Classification and pathophysiological implications. Cell Death Differ..

[29] Galluzzi L., Bravo-San Pedro J.M., Vitale I., Aaronson S.A., Abrams J.M., Adam D., Alnemri E.S., Altucci L., Andrews D., Annicchiarico-Petruzzelli M. (2015). Essential versus accessory aspects of cell death: Recommendations of the NCCD 2015. Cell Death Differ..

[30] Galluzzi L., Vitale I., Aaronson S.A., Abrams J.M., Adam D., Agostinis P., Alnemri E.S., Altucci L., Amelio I., Andrews D.W. (2018). Molecular mechanisms of cell death: Recommendations of the Nomenclature Committee on Cell Death 2018. Cell Death Differ..

[31] Maltese W.A., Overmeyer J.H. (2014). Methuosis: Nonapoptotic cell death associated with vacuolization of macropinosome and endosome compartments. Am. J. Pathol..

[32] Fontana F., Raimondi M., Marzagalli M., Di Domizio A., Limonta P. (2020). The emerging role of paraptosis in tumor cell biology: Perspectives for cancer prevention and therapy with natural compounds. Biochim. Et Biophys. Acta. Rev. Cancer.

[33] Liu Y., Shoji-Kawata S., Sumpter R.M., Wei Y., Ginet V., Zhang L., Posner B., Tran K.A., Green D.R., Xavier R.J. (2013). Autosis is a Na+,K+-ATPase-regulated form of cell death triggered by autophagy-inducing peptides, starvation, and hypoxia-ischemia. Proc. Natl. Acad. Sci. USA.

[34] Liu J., Kuang F., Kang R., Tang D. (2020). Alkaliptosis: A new weapon for cancer therapy. Cancer Gene Ther..

[35] Holze C., Michaudel C., Mackowiak C., Haas D.A., Benda C., Hubel P., Pennemann F.L., Schnepf D., Wettmarshausen J., Braun M. (2018). Oxeiptosis, a ROS-induced caspase-independent apoptosis-like cell-death pathway. Nat. Immunol..

[36] Tang D., Chen X., Kroemer G. (2022). Cuproptosis: A copper-triggered modality of mitochondrial cell death. Cell Res..

[37] Ciesielski H.M., Nishida H., Takano T., Fukuhara A., Otani T., Ikegawa Y., Okada M., Nishimura T., Furuse M., Yoo S.K. (2022). Erebosis, a new cell death mechanism during homeostatic turnover of gut enterocytes. PLoS Biol..

[38] Dolma S., Lessnick S.L., Hahn W.C., Stockwell B.R. (2003). Identification of genotype-selective antitumor agents using synthetic lethal chemical screening in engineered human tumor cells. Cancer Cell.

[39] Yang W.S., Stockwell B.R. (2008). Synthetic lethal screening identifies compounds activating iron-dependent, nonapoptotic cell death in oncogenic-RAS-harboring cancer cells. Chem. Biol..

[40] Yagoda N., von Rechenberg M., Zaganjor E., Bauer A.J., Yang W.S., Fridman D.J., Wolpaw A.J., Smukste I., Peltier J.M., Boniface J.J. (2007). RAS-RAF-MEK-dependent oxidative cell death involving voltage-dependent anion channels. Nature.

[41] Seiler A., Schneider M., Forster H., Roth S., Wirth E.K., Culmsee C., Plesnila N., Kremmer E., Radmark O., Wurst W. (2008). Glutathione peroxidase 4 senses and translates oxidative stress into 12/15-lipoxygenase dependent- and AIF-mediated cell death. Cell Metab..

[42] Banjac A., Perisic T., Sato H., Seiler A., Bannai S., Weiss N., Kolle P., Tschoep K., Issels R.D., Daniel P.T. (2008). The cystine/cysteine cycle: A redox cycle regulating susceptibility versus resistance to cell death. Oncogene.

[43] Dixon S.J., Lemberg K.M., Lamprecht M.R., Skouta R., Zaitsev E.M., Gleason C.E., Patel D.N., Bauer A.J., Cantley A.M., Yang W.S. (2012). Ferroptosis: An iron-dependent form of nonapoptotic cell death. Cell.

[44] Tang D., Chen X., Kang R., Kroemer G. (2021). Ferroptosis: Molecular mechanisms and health implications. Cell Res..

[45] Tan S., Schubert D., Maher P. (2001). Oxytosis: A novel form of programmed cell death. Curr. Top. Med. Chem..

[46] Maher P., Currais A., Schubert D. (2020). Using the Oxytosis/Ferroptosis Pathway to Understand and Treat Age-Associated Neurodegenerative Diseases. Cell Chem. Biol..

[47] Henke N., Albrecht P., Bouchachia I., Ryazantseva M., Knoll K., Lewerenz J., Kaznacheyeva E., Maher P., Methner A. (2013). The plasma membrane channel ORAI1 mediates detrimental calcium influx caused by endogenous oxidative stress. Cell Death Dis..

[48] Feng H., Stockwell B.R. (2018). Unsolved mysteries: How does lipid peroxidation cause ferroptosis?. PLoS Biol..

[49] Wang L., Chen X., Yan C. (2022). Ferroptosis: An emerging therapeutic opportunity for cancer. Genes Dis..

[50] Gout P.W., Buckley A.R., Simms C.R., Bruchovsky N. (2001). Sulfasalazine, a potent suppressor of lymphoma growth by inhibition of the x(c)- cystine transporter: A new action for an old drug. Leukemia.

[51] Louandre C., Ezzoukhry Z., Godin C., Barbare J.C., Maziere J.C., Chauffert B., Galmiche A. (2013). Iron-dependent cell death of hepatocellular carcinoma cells exposed to sorafenib. Int. J. Cancer.

[52] Yang W.S., SriRamaratnam R., Welsch M.E., Shimada K., Skouta R., Viswanathan V.S., Cheah J.H., Clemons P.A., Shamji A.F., Clish C.B. (2014). Regulation of ferroptotic cancer cell death by GPX4. Cell.

[53] Dixon S.J., Winter G.E., Musavi L.S., Lee E.D., Snijder B., Rebsamen M., Superti-Furga G., Stockwell B.R. (2015). Human Haploid Cell Genetics Reveals Roles for Lipid Metabolism Genes in Nonapoptotic Cell Death. ACS Chem. Biol..

[54] Shimada K., Skouta R., Kaplan A., Yang W.S., Hayano M., Dixon S.J., Brown L.M., Valenzuela C.A., Wolpaw A.J., Stockwell B.R. (2016). Global survey of cell death mechanisms reveals metabolic regulation of ferroptosis. Nat. Chem. Biol..

[55] Gaschler M.M., Andia A.A., Liu H., Csuka J.M., Hurlocker B., Vaiana C.A., Heindel D.W., Zuckerman D.S., Bos P.H., Reznik E. (2018). FINO(2) initiates ferroptosis through GPX4 inactivation and iron oxidation. Nat. Chem. Biol..

[56] Liptay S., Fulda S., Schanbacher M., Bourteele S., Ferri K.F., Kroemer G., Adler G., Debatin K.M., Schmid R.M. (2002). Molecular mechanisms of sulfasalazine-induced T-cell apoptosis. Br. J. Pharmacol..

[57] Zheng J., Sato M., Mishima E., Sato H., Proneth B., Conrad M. (2021). Sorafenib fails to trigger ferroptosis across a wide range of cancer cell lines. Cell Death Dis..

[58] Wang D., Peng Y., Xie Y., Zhou B., Sun X., Kang R., Tang D. (2016). Antiferroptotic activity of non-oxidative dopamine. Biochem. Biophys. Res. Commun..

[59] Cardoso B.R., Hare D.J., Bush A.I., Roberts B.R. (2017). Glutathione peroxidase 4: A new player in neurodegeneration?. Mol. Psychiatry.

[60] Zhang Y., Zhang X., Wee Yong V., Xue M. (2022). Vildagliptin improves neurological function by inhibiting apoptosis and ferroptosis following intracerebral hemorrhage in mice. Neurosci. Lett..

[61] Gaschler M.M., Stockwell B.R. (2017). Lipid peroxidation in cell death. Biochem. Biophys. Res. Commun..

[62] Shah R., Shchepinov M.S., Pratt D.A. (2018). Resolving the Role of Lipoxygenases in the Initiation and Execution of Ferroptosis. ACS Cent. Sci..

[63] Wang H., Liu C., Zhao Y., Gao G. (2020). Mitochondria regulation in ferroptosis. Eur. J. Cell Biol..

[64] Gaschler M.M., Hu F., Feng H., Linkermann A., Min W., Stockwell B.R. (2018). Determination of the Subcellular Localization and Mechanism of Action of Ferrostatins in Suppressing Ferroptosis. ACS Chem. Biol..

[65] Jelinek A., Heyder L., Daude M., Plessner M., Krippner S., Grosse R., Diederich W.E., Culmsee C. (2018). Mitochondrial rescue prevents glutathione peroxidase-dependent ferroptosis. Free Radic. Biol. Med..

[66] Wang Y.Q., Chang S.Y., Wu Q., Gou Y.J., Jia L., Cui Y.M., Yu P., Shi Z.H., Wu W.S., Gao G. (2016). The Protective Role of Mitochondrial Ferritin on Erastin-Induced Ferroptosis. Front. Aging Neurosci..

[67] Hou W., Xie Y., Song X., Sun X., Lotze M.T., Zeh H.J., Kang R., Tang D. (2016). Autophagy promotes ferroptosis by degradation of ferritin. Autophagy.

[68] Dalleau S., Baradat M., Gueraud F., Huc L. (2013). Cell death and diseases related to oxidative stress: 4-hydroxynonenal (HNE) in the balance. Cell Death Differ..

[69] Zarkovic N., Cipak A., Jaganjac M., Borovic S., Zarkovic K. (2013). Pathophysiological relevance of aldehydic protein modifications. J. Proteom..

[70] Luo L., Wang H., Tian W., Li X., Zhu Z., Huang R., Luo H. (2021). Targeting ferroptosis-based cancer therapy using nanomaterials: Strategies and applications. Theranostics.

[71] Xie Y., Hou W., Song X., Yu Y., Huang J., Sun X., Kang R., Tang D. (2016). Ferroptosis: Process and function. Cell Death Differ..

[72] Goncalves D.A., Jasiulionis M.G., Melo F.H.M. (2021). The Role of the BH4 Cofactor in Nitric Oxide Synthase Activity and Cancer Progression: Two Sides of the Same Coin. Int. J. Mol. Sci..

[73] Song R., Li T., Ye J., Sun F., Hou B., Saeed M., Gao J., Wang Y., Zhu Q., Xu Z. (2021). Acidity-Activatable Dynamic Nanoparticles Boosting Ferroptotic Cell Death for Immunotherapy of Cancer. Adv. Mater..

[74] Lang X., Green M.D., Wang W., Yu J., Choi J.E., Jiang L., Liao P., Zhou J., Zhang Q., Dow A. (2019). Radiotherapy and Immunotherapy Promote Tumoral Lipid Oxidation and Ferroptosis via Synergistic Repression of SLC7A11. Cancer Discov..

[75] Efimova I., Catanzaro E., Van der Meeren L., Turubanova V.D., Hammad H., Mishchenko T.A., Vedunova M.V., Fimognari C., Bachert C., Coppieters F. (2020). Vaccination with early ferroptotic cancer cells induces efficient antitumor immunity. J. Immunother. Cancer.

[76] Wiernicki B., Maschalidi S., Pinney J., Adjemian S., Vanden Berghe T., Ravichandran K.S., Vandenabeele P. (2022). Cancer cells dying from ferroptosis impede dendritic cell-mediated anti-tumor immunity. Nat. Commun..

[77] Luo X., Gong H.B., Gao H.Y., Wu Y.P., Sun W.Y., Li Z.Q., Wang G., Liu B., Liang L., Kurihara H. (2021). Oxygenated phosphatidylethanolamine navigates phagocytosis of ferroptotic cells by interacting with TLR2. Cell Death Differ..

[78] Agmon E., Solon J., Bassereau P., Stockwell B.R. (2018). Modeling the effects of lipid peroxidation during ferroptosis on membrane properties. Sci. Rep..

[79] Friedmann Angeli J.P., Krysko D.V., Conrad M. (2019). Ferroptosis at the crossroads of cancer-acquired drug resistance and immune evasion. Nat. Rev. Cancer.

[80] Rothe T., Gruber F., Uderhardt S., Ipseiz N., Rossner S., Oskolkova O., Bluml S., Leitinger N., Bicker W., Bochkov V.N. (2015). 12/15-Lipoxygenase-mediated enzymatic lipid oxidation regulates DC maturation and function. J. Clin. Investig..

[81] Ma X., Xiao L., Liu L., Ye L., Su P., Bi E., Wang Q., Yang M., Qian J., Yi Q. (2021). CD36-mediated ferroptosis dampens intratumoral CD8(+) T cell effector function and impairs their antitumor ability. Cell Metab..

[82] Dai E., Han L., Liu J., Xie Y., Zeh H.J., Kang R., Bai L., Tang D. (2020). Ferroptotic damage promotes pancreatic tumorigenesis through a TMEM173/STING-dependent DNA sensor pathway. Nat. Commun..

[83] Dai E., Han L., Liu J., Xie Y., Kroemer G., Klionsky D.J., Zeh H.J., Kang R., Wang J., Tang D. (2020). Autophagy-dependent ferroptosis drives tumor-associated macrophage polarization via release and uptake of oncogenic KRAS protein. Autophagy.

[84] Jiang Z., Lim S.O., Yan M., Hsu J.L., Yao J., Wei Y., Chang S.S., Yamaguchi H., Lee H.H., Ke B. (2021). TYRO3 induces anti-PD-1/PD-L1 therapy resistance by limiting innate immunity and tumoral ferroptosis. J. Clin. Investig..

[85] Siliakus M.F., van der Oost J., Kengen S.W.M. (2017). Adaptations of archaeal and bacterial membranes to variations in temperature, pH and pressure. Extrem. Life Under Extrem. Cond..

[86] Chaudhary A., Ketkar O.A., Irfan S., Rana V., Rahi P., Deshmukh R., Kaur J., Dhar H. (2022). Genomic Insights into Omega-3 Polyunsaturated Fatty Acid Producing Shewanella sp. N2AIL from Fish Gut. Biology.

[87] Moravec A.R., Siv A.W., Hobby C.R., Lindsay E.N., Norbash L.V., Shults D.J., Symes S.J.K., Giles D.K. (2017). Exogenous Polyunsaturated Fatty Acids Impact Membrane Remodeling and Affect Virulence Phenotypes among Pathogenic Vibrio Species. Appl. Environ. Microbiol..

[88] Hill S., Lamberson C.R., Xu L., To R., Tsui H.S., Shmanai V.V., Bekish A.V., Awad A.M., Marbois B.N., Cantor C.R. (2012). Small amounts of isotope-reinforced polyunsaturated fatty acids suppress lipid autoxidation. Free Radic. Biol. Med..

[89] Distefano A.M., Martin M.V., Cordoba J.P., Bellido A.M., D’Ippolito S., Colman S.L., Soto D., Roldan J.A., Bartoli C.G., Zabaleta E.J. (2017). Heat stress induces ferroptosis-like cell death in plants. J. Cell Biol..

[90] Jiang L., Kon N., Li T., Wang S.J., Su T., Hibshoosh H., Baer R., Gu W. (2015). Ferroptosis as a p53-mediated activity during tumour suppression. Nature.

[91] Wang S.J., Li D., Ou Y., Jiang L., Chen Y., Zhao Y., Gu W. (2016). Acetylation Is Crucial for p53-Mediated Ferroptosis and Tumor Suppression. Cell Rep..

[92] Zhang Y., Shi J., Liu X., Feng L., Gong Z., Koppula P., Sirohi K., Li X., Wei Y., Lee H. (2018). BAP1 links metabolic regulation of ferroptosis to tumour suppression. Nat. Cell Biol..

[93] Gao M., Yi J., Zhu J., Minikes A.M., Monian P., Thompson C.B., Jiang X. (2019). Role of Mitochondria in Ferroptosis. Mol. Cell.

[94] Dixon S.J. (2017). Ferroptosis: Bug or feature?. Immunol. Rev..

[95] Tang D., Kepp O., Kroemer G. (2020). Ferroptosis becomes immunogenic: Implications for anticancer treatments. Oncoimmunology.

[96] Kagan V.E., Mao G., Qu F., Angeli J.P., Doll S., Croix C.S., Dar H.H., Liu B., Tyurin V.A., Ritov V.B. (2017). Oxidized arachidonic and adrenic PEs navigate cells to ferroptosis. Nat. Chem. Biol..

[97] Drummen G.P., van Liebergen L.C., Op den Kamp J.A., Post J.A. (2002). C11-BODIPY(581/591), an oxidation-sensitive fluorescent lipid peroxidation probe: (micro)spectroscopic characterization and validation of methodology. Free Radic. Biol. Med..

[98] Aron A.T., Loehr M.O., Bogena J., Chang C.J. (2016). An Endoperoxide Reactivity-Based FRET Probe for Ratiometric Fluorescence Imaging of Labile Iron Pools in Living Cells. J. Am. Chem. Soc..

[99] Shi L., Guan Q., Gao X., Jin X., Xu L., Shen J., Wu C., Zhu X., Zhang C. (2018). Reaction-Based Color-Convertible Fluorescent Probe for Ferroptosis Identification. Anal. Chem..

[100] Lorendeau D., Christen S., Rinaldi G., Fendt S.M. (2015). Metabolic control of signalling pathways and metabolic auto-regulation. Biol. Cell.

[101] Luo J. (2011). Cancer’s sweet tooth for serine. Breast Cancer Res. BCR.

[102] Patil M.D., Bhaumik J., Babykutty S., Banerjee U.C., Fukumura D. (2016). Arginine dependence of tumor cells: Targeting a chink in cancer’s armor. Oncogene.

[103] Wise D.R., Thompson C.B. (2010). Glutamine addiction: A new therapeutic target in cancer. Trends Biochem. Sci..

[104] Altea-Manzano P., Cuadros A.M., Broadfield L.A., Fendt S.M. (2020). Nutrient metabolism and cancer in the in vivo context: A metabolic game of give and take. EMBO Rep..

[105] Fendt S.M., Frezza C., Erez A. (2020). Targeting Metabolic Plasticity and Flexibility Dynamics for Cancer Therapy. Cancer Discov..

[106] Grasmann G., Mondal A., Leithner K. (2021). Flexibility and Adaptation of Cancer Cells in a Heterogenous Metabolic Microenvironment. Int. J. Mol. Sci..

[107] Tasdogan A., Faubert B., Ramesh V., Ubellacker J.M., Shen B., Solmonson A., Murphy M.M., Gu Z., Gu W., Martin M. (2020). Metabolic heterogeneity confers differences in melanoma metastatic potential. Nature.

[108] Bhutia Y.D., Babu E., Ramachandran S., Ganapathy V. (2015). Amino Acid transporters in cancer and their relevance to “glutamine addiction”: Novel targets for the design of a new class of anticancer drugs. Cancer Res..

[109] Bannai S., Ishii T. (1988). A novel function of glutamine in cell culture: Utilization of glutamine for the uptake of cystine in human fibroblasts. J. Cell. Physiol..

[110] Shin C.S., Mishra P., Watrous J.D., Carelli V., D’Aurelio M., Jain M., Chan D.C. (2017). The glutamate/cystine xCT antiporter antagonizes glutamine metabolism and reduces nutrient flexibility. Nat. Commun..

[111] Koppula P., Zhang Y., Shi J., Li W., Gan B. (2017). The glutamate/cystine antiporter SLC7A11/xCT enhances cancer cell dependency on glucose by exporting glutamate. J. Biol. Chem..

[112] Koppula P., Zhuang L., Gan B. (2021). Cystine transporter SLC7A11/xCT in cancer: Ferroptosis, nutrient dependency, and cancer therapy. Protein Cell.

[113] Makker V., MacKay H., Ray-Coquard I., Levine D.A., Westin S.N., Aoki D., Oaknin A. (2021). Endometrial cancer. Nat. Rev. Dis. Primers.

[114] Renehan A.G., Zwahlen M., Egger M. (2015). Adiposity and cancer risk: New mechanistic insights from epidemiology. Nat. Rev. Cancer.

[115] Rodriguez A.C., Vahrenkamp J.M., Berrett K.C., Clark K.A., Guillen K.P., Scherer S.D., Yang C.H., Welm B.E., Janat-Amsbury M.M., Graves B.J. (2020). ETV4 Is Necessary for Estrogen Signaling and Growth in Endometrial Cancer Cells. Cancer Res..

[116] Qi Y., Tan M., Zheng M., Jin S., Wang H., Liu J., Wang P., Nie X., Gao L., Lin B. (2020). Estrogen/estrogen receptor promotes the proliferation of endometrial carcinoma cells by enhancing hMOF expression. Jpn. J. Clin. Oncol..

[117] Tian H., Gao Z., Wang G., Li H., Zheng J. (2016). Estrogen potentiates reactive oxygen species (ROS) tolerance to initiate carcinogenesis and promote cancer malignant transformation. Tumour Biol. J. Int. Soc. Oncodevelopmental Biol. Med..

[118] Vekic J., Zeljkovic A., Stefanovic A., Jelic-Ivanovic Z., Spasojevic-Kalimanovska V. (2019). Obesity and dyslipidemia. Metab. Clin. Exp..

[119] Vercesi A.E., Castilho R.F., Kowaltowski A.J., Oliveira H.C. (2007). Mitochondrial energy metabolism and redox state in dyslipidemias. IUBMB Life.

[120] Ma X., Pham V.T., Mori H., MacDougald O.A., Shah Y.M., Bodary P.F. (2017). Iron elevation and adipose tissue remodeling in the epididymal depot of a mouse model of polygenic obesity. PLoS ONE.

[121] Khabaz M.N., Qureshi I.A., Al-Maghrabi J.A. (2019). GLUT 1 expression is a supportive mean in predicting prognosis and survival estimates of endometrial carcinoma. Ginekol. Pol..

[122] Marshall A.D., van Geldermalsen M., Otte N.J., Lum T., Vellozzi M., Thoeng A., Pang A., Nagarajah R., Zhang B., Wang Q. (2017). ASCT2 regulates glutamine uptake and cell growth in endometrial carcinoma. Oncogenesis.

[123] Fang X., Zhang T., Chen Z. (2023). Solute Carrier Family 7 Member 11 (SLC7A11) is a Potential Prognostic Biomarker in Uterine Corpus Endometrial Carcinoma. Int. J. Gen. Med..

[124] Byrne F.L., Poon I.K., Modesitt S.C., Tomsig J.L., Chow J.D., Healy M.E., Baker W.D., Atkins K.A., Lancaster J.M., Marchion D.C. (2014). Metabolic vulnerabilities in endometrial cancer. Cancer Res..

[125] DeWaal D., Nogueira V., Terry A.R., Patra K.C., Jeon S.M., Guzman G., Au J., Long C.P., Antoniewicz M.R., Hay N. (2018). Hexokinase-2 depletion inhibits glycolysis and induces oxidative phosphorylation in hepatocellular carcinoma and sensitizes to metformin. Nat. Commun..

[126] Wang L., Huang Q., Lin Q., Chen L., Shi Q. (2021). Knockdown of long non-coding RNA small nucleolar RNA host gene 9 or hexokinase 2 both suppress endometrial cancer cell proliferation and glycolysis. J. Obstet. Gynaecol. Res..

[127] Zhang G., Ma A., Jin Y., Pan G., Wang C. (2019). LncRNA SNHG16 induced by TFAP2A modulates glycolysis and proliferation of endometrial carcinoma through miR-490-3p/HK2 axis. Am. J. Transl. Res..

[128] Han X., Ren C., Yang T., Qiao P., Wang L., Jiang A., Meng Y., Liu Z., Du Y., Yu Z. (2019). Negative regulation of AMPKalpha1 by PIM2 promotes aerobic glycolysis and tumorigenesis in endometrial cancer. Oncogene.

[129] Yu S., Chen Z., Zeng X., Chen X., Gu Z. (2019). Advances in nanomedicine for cancer starvation therapy. Theranostics.

[130] Cheng H., Jiang X.Y., Zheng R.R., Zuo S.J., Zhao L.P., Fan G.L., Xie B.R., Yu X.Y., Li S.Y., Zhang X.Z. (2019). A biomimetic cascade nanoreactor for tumor targeted starvation therapy-amplified chemotherapy. Biomaterials.

[131] Li J., Li Y., Wang Y., Ke W., Chen W., Wang W., Ge Z. (2017). Polymer Prodrug-Based Nanoreactors Activated by Tumor Acidity for Orchestrated Oxidation/Chemotherapy. Nano Lett..

[132] Yang J., Ma S., Xu R., Wei Y., Zhang J., Zuo T., Wang Z., Deng H., Yang N., Shen Q. (2021). Smart biomimetic metal organic frameworks based on ROS-ferroptosis-glycolysis regulation for enhanced tumor chemo-immunotherapy. J. Control. Release Off. J. Control. Release Soc..

[133] Guo H., Li W., Pan G., Wang C., Li D., Liu N., Sheng X., Yuan L. (2023). The Glutaminase Inhibitor Compound 968 Exhibits Potent In vitro and In vivo Anti-tumor Effects in Endometrial Cancer. Anti-Cancer Agents Med. Chem..

[134] Basuli D., Tesfay L., Deng Z., Paul B., Yamamoto Y., Ning G., Xian W., McKeon F., Lynch M., Crum C.P. (2017). Iron addiction: A novel therapeutic target in ovarian cancer. Oncogene.

[135] Greenshields A.L., Shepherd T.G., Hoskin D.W. (2017). Contribution of reactive oxygen species to ovarian cancer cell growth arrest and killing by the anti-malarial drug artesunate. Mol. Carcinog..

[136] Cancer Genome Atlas Research N. (2011). Integrated genomic analyses of ovarian carcinoma. Nature.

[137] Zhang Y., Xia M., Zhou Z., Hu X., Wang J., Zhang M., Li Y., Sun L., Chen F., Yu H. (2021). p53 Promoted Ferroptosis in Ovarian Cancer Cells Treated with Human Serum Incubated-Superparamagnetic Iron Oxides. Int. J. Nanomed..

[138] Hong T., Lei G., Chen X., Li H., Zhang X., Wu N., Zhao Y., Zhang Y., Wang J. (2021). PARP inhibition promotes ferroptosis via repressing SLC7A11 and synergizes with ferroptosis inducers in BRCA-proficient ovarian cancer. Redox Biol..

[139] Seborova K., Vaclavikova R., Soucek P., Elsnerova K., Bartakova A., Cernaj P., Bouda J., Rob L., Hruda M., Dvorak P. (2019). Association of ABC gene profiles with time to progression and resistance in ovarian cancer revealed by bioinformatics analyses. Cancer Med..

[140] Jiang X., Shi M., Sui M., Yuan Y., Zhang S., Xia Q., Zhao K. (2021). Oleanolic acid inhibits cervical cancer Hela cell proliferation through modulation of the ACSL4 ferroptosis signaling pathway. Biochem. Biophys. Res. Commun..

[141] Wang C., Zeng J., Li L.J., Xue M., He S.L. (2021). Cdc25A inhibits autophagy-mediated ferroptosis by upregulating ErbB2 through PKM2 dephosphorylation in cervical cancer cells. Cell Death Dis..

[142] Ye R.R., Chen B.C., Lu J.J., Ma X.R., Li R.T. (2021). Phosphorescent rhenium(I) complexes conjugated with artesunate: Mitochondrial targeting and apoptosis-ferroptosis dual induction. J. Inorg. Biochem..

[143] Ma Y., Wang W., Idowu M.O., Oh U., Wang X.Y., Temkin S.M., Fang X. (2018). Ovarian Cancer Relies on Glucose Transporter 1 to Fuel Glycolysis and Growth: Anti-Tumor Activity of BAY-876. Cancers.

[144] Xintaropoulou C., Ward C., Wise A., Queckborner S., Turnbull A., Michie C.O., Williams A.R.W., Rye T., Gourley C., Langdon S.P. (2018). Expression of glycolytic enzymes in ovarian cancers and evaluation of the glycolytic pathway as a strategy for ovarian cancer treatment. BMC Cancer.

[145] Pasto A., Pagotto A., Pilotto G., De Paoli A., De Salvo G.L., Baldoni A., Nicoletto M.O., Ricci F., Damia G., Bellio C. (2017). Resistance to glucose starvation as metabolic trait of platinum-resistant human epithelial ovarian cancer cells. Oncotarget.

[146] Zhang X.Y., Zhang M., Cong Q., Zhang M.X., Zhang M.Y., Lu Y.Y., Xu C.J. (2018). Hexokinase 2 confers resistance to cisplatin in ovarian cancer cells by enhancing cisplatin-induced autophagy. Int. J. Biochem. Cell Biol..

[147] Jiang Y.X., Siu M.K.Y., Wang J.J., Leung T.H.Y., Chan D.W., Cheung A.N.Y., Ngan H.Y.S., Chan K.K.L. (2022). PFKFB3 Regulates Chemoresistance, Metastasis and Stemness via IAP Proteins and the NF-kappaB Signaling Pathway in Ovarian Cancer. Front. Oncol..

[148] Chao T.K., Huang T.S., Liao Y.P., Huang R.L., Su P.H., Shen H.Y., Lai H.C., Wang Y.C. (2017). Pyruvate kinase M2 is a poor prognostic marker of and a therapeutic target in ovarian cancer. PLoS ONE.

[149] Boran N., Kayikcioglu F., Yalvac S., Tulunay G., Ekinci U., Kose M.F. (2000). Significance of serum and peritoneal fluid lactate dehydrogenase levels in ovarian cancer. Gynecol. Obstet. Investig..

[150] Wu Y., Zhang X., Wang Z., Zheng W., Cao H., Shen W. (2022). Targeting oxidative phosphorylation as an approach for the treatment of ovarian cancer. Front. Oncol..

[151] Hudson C.D., Savadelis A., Nagaraj A.B., Joseph P., Avril S., DiFeo A., Avril N. (2016). Altered glutamine metabolism in platinum resistant ovarian cancer. Oncotarget.

[152] Kim B.H., Chang J.H. (2019). Differential effect of GLUT1 overexpression on survival and tumor immune microenvironment of human papilloma virus type 16-positive and -negative cervical cancer. Sci. Rep..

[153] Mondal S., Roy D., Sarkar Bhattacharya S., Jin L., Jung D., Zhang S., Kalogera E., Staub J., Wang Y., Xuyang W. (2019). Therapeutic targeting of PFKFB3 with a novel glycolytic inhibitor PFK158 promotes lipophagy and chemosensitivity in gynecologic cancers. Int. J. Cancer.

[154] Arizmendi-Izazaga A., Navarro-Tito N., Jimenez-Wences H., Mendoza-Catalan M.A., Martinez-Carrillo D.N., Zacapala-Gomez A.E., Olea-Flores M., Dircio-Maldonado R., Torres-Rojas F.I., Soto-Flores D.G. (2021). Metabolic Reprogramming in Cancer: Role of HPV 16 Variants. Pathogens.

[155] Xiao X., Shan H., Niu Y., Wang P., Li D., Zhang Y., Wang J., Wu Y., Jiang H. (2022). TMPRSS2 Serves as a Prognostic Biomarker and Correlated with Immune Infiltrates in Breast Invasive Cancer and Lung Adenocarcinoma. Front. Mol. Biosci..

[156] Thumser-Henner C., Oeck S., Kalmbach S., Forster J., Kindl F., Sak A., Schramm A., Schuler M. (2022). Syntaxin 18 regulates the DNA damage response and epithelial-to-mesenchymal transition to promote radiation resistance of lung cancer. Cell Death Dis..

[157] Gu J., Dong L., Wang Y., Nie W., Liu W., Zhao J.A. (2021). LINC01224 promotes colorectal cancer progression through targeting miR-485-5p/MYO6 axis. World J. Surg. Oncol..

[158] Lu H., Shi C., Liu X., Liang C., Yang C., Wan X., Li L., Liu Y. (2021). Identification of ZG16B as a prognostic biomarker in breast cancer. Open Med..

[159] Escudero-Paniagua B., Bartolome R.A., Rodriguez S., De Los Rios V., Pintado L., Jaen M., Lafarga M., Fernandez-Acenero M.J., Casal J.I. (2020). PAUF/ZG16B promotes colorectal cancer progression through alterations of the mitotic functions and the Wnt/beta-catenin pathway. Carcinogenesis.

[160] Hou X., Tang L., Li X., Xiong F., Mo Y., Jiang X., Deng X., Peng M., Wu P., Zhao M. (2021). Potassium Channel Protein KCNK6 Promotes Breast Cancer Cell Proliferation, Invasion, and Migration. Front. Cell Dev. Biol..

[161] Correa R.G., Krajewska M., Ware C.F., Gerlic M., Reed J.C. (2014). The NLR-related protein NWD1 is associated with prostate cancer and modulates androgen receptor signaling. Oncotarget.

[162] Wang H., Sun Q., Wu Y., Wang L., Zhou C., Ma W., Zhang Y., Wang S., Zhang S. (2015). Granzyme M expressed by tumor cells promotes chemoresistance and EMT in vitro and metastasis in vivo associated with STAT3 activation. Oncotarget.

[163] Przygodzka P., Soboska K., Sochacka E., Pacholczyk M., Braun M., Kassassir H., Papiewska-Pajak I., Kielbik M., Boncela J. (2022). Neuromedin U secreted by colorectal cancer cells promotes a tumour-supporting microenvironment. Cell Commun. Signal. CCS.

[164] Ou Y., Wang S.J., Li D., Chu B., Gu W. (2016). Activation of SAT1 engages polyamine metabolism with p53-mediated ferroptotic responses. Proc. Natl. Acad. Sci. USA.

[165] Mou Y., Zhang L., Liu Z., Song X. (2022). Abundant expression of ferroptosis-related SAT1 is related to unfavorable outcome and immune cell infiltration in low-grade glioma. BMC Cancer.

[166] Yang P., Lu J., Zhang P., Zhang S. (2023). Comprehensive Analysis of Prognosis and Immune Landscapes Based on Lipid-Metabolism- and Ferroptosis-Associated Signature in Uterine Corpus Endometrial Carcinoma. Diagnostics.

[167] Zhang Y., Guo S., Wang S., Li X., Hou D., Li H., Wang L., Xu Y., Ma B., Wang H. (2021). LncRNA OIP5-AS1 inhibits ferroptosis in prostate cancer with long-term cadmium exposure through miR-128-3p/SLC7A11 signaling. Ecotoxicol. Environ. Saf..

[168] Wang M., Mao C., Ouyang L., Liu Y., Lai W., Liu N., Shi Y., Chen L., Xiao D., Yu F. (2020). Correction to: Long noncoding RNA LINC00336 inhibits ferroptosis in lung cancer by functioning as a competing endogenous RNA. Cell Death Differ..

[169] Luo W., Wang J., Xu W., Ma C., Wan F., Huang Y., Yao M., Zhang H., Qu Y., Ye D. (2021). LncRNA RP11-89 facilitates tumorigenesis and ferroptosis resistance through PROM2-activated iron export by sponging miR-129-5p in bladder cancer. Cell Death Dis..

[170] Peng J., Hao Y., Rao B., Zhang Z. (2021). A ferroptosis-related lncRNA signature predicts prognosis in ovarian cancer patients. Transl. Cancer Res..

[171] Qin A., Qian Q., Cui X., Bai W. (2023). Ferroptosis-related lncRNA model based on CFAP58-DT for predicting prognosis and immunocytes infiltration in endometrial cancer. Ann. Transl. Med..

[172] Liu Z., Zhao Q., Zuo Z.X., Yuan S.Q., Yu K., Zhang Q., Zhang X., Sheng H., Ju H.Q., Cheng H. (2020). Systematic Analysis of the Aberrances and Functional Implications of Ferroptosis in Cancer. iScience.

[173] Zhang K., Li H., Yan Y., Zang Y., Li K., Wang Y., Xue F. (2019). Identification of key genes and pathways between type I and type II endometrial cancer using bioinformatics analysis. Oncol. Lett..

